# Alexithymia and its association with burnout, depression and family support among Greek nursing staff

**DOI:** 10.1186/1478-4491-7-72

**Published:** 2009-08-11

**Authors:** Dionisios Bratis, Athanasios Tselebis, Christos Sikaras, Aikaterini Moulou, Konstantinos Giotakis, Emmanuel Zoumakis, Ioannis Ilias

**Affiliations:** 1 Sotiria General Hospital of Chest Diseases, Athens, Greece; 2 First Department of Pediatrics, University of Athens Medical School, Athens, Greece; 3 Endocrine Department, Elena Venizelou Hospital, Athens, Greece

## Abstract

**Background:**

Few studies have examined the relation between alexithymia (i.e. the inability to recognize and verbalize emotions) and professional burnout. Considering the absence of relevant studies in the Greek scientific literature, the aim of this work was to examine the associations of alexithymia with the three facets of professional burnout, the perception of family support and depression in nursing personnel.

**Methods:**

The study was performed in one of the largest hospitals in Greece and included 95 nurses. Assessments of alexithymia, burnout, depression and family support were made by means of the Toronto Alexithymia Scale, the Maslach Burnout Inventory, the Beck Depression Inventory and the Julkunen Family Support Scale, respectively. Student's t-test, Pearson's correlation and stepwise linear regression were used for the evaluation of data.

**Results:**

Alexithymia was correlated positively with depression, emotional exhaustion and depersonalization, and negatively with sense of family support and personal achievement. Additionally, family support was correlated positively with personal achievement and negatively with depression.

**Conclusion:**

In the scientific literature there is a debate as to whether alexithymia is a stable personality characteristic or if it is dependent on symptoms of mental disorders. We tried to interpret the associations of alexithymia with professional burnout, depressive symptoms and family support. From this study it appears very likely that alexithymia is directly associated with depression and personal achievement, but also - indirectly - with the sense of family support.

## Background

Sifneos introduced the notion of alexithymia as the inability to recognize and verbalize emotions [1,2]. Newer studies on alexithymia defined it as weakness in the determination and expression of emotions; moreover, alexithymia encompasses externally directed thought and limited imaginative faculty [3].

Alexithymia appears to be positively associated with depression in the general population [4] and has been shown to be associated with several diseases [5]; in characteristically alexithymic subjects the favourable effect of family and social support on depressive symptomatology is diminished [6,7]. Alexithymia is conceptualized as a stable personality trait; some studies have associated it with psychopathological disorders (such as depression or anxiety) or with somatic diseases [8].

The notion of professional burnout was introduced by Freudenberger, who described overstrain symptoms that he observed in professionals and volunteers in the mental health sector [9]. The most widely accepted definition of burnout was formulated by Maslach, who described it as a mental syndrome (along with bodily exhaustion) that develops in people who have a professional relationship with other persons [10]: the worker loses the interest and positive sentiments that he/she had for patients or customers and develops a negative self-image.

Depression, which is common in mental health workers compared with the general population [11] in a number of reports, is correlated positively with professional burnout [12,13].

Family support refers to the sense of support that an individual perceives he or she receives from his or her familial environment; it constitutes an important element of social support, particularly in the Greek population, with its close-knit families [14]. The positive effect of family support becomes particularly obvious in patients with chronic diseases, as in the case of diabetes [14,15]. Research stresses the negative cross-correlation that is observed between family support and depression [6], while a recent study reports the existence of negative cross-correlation between family support and professional burnout [16].

Few studies have examined the relationship between alexithymia and professional burnout [17,18]. The findings suggest that alexithymia is significantly associated with burnout even when controlled for confounding factors [18].

Considering the absence of relevant studies in the Greek scientific literature, the aim of this work was to examine the associations of alexithymia with professional burnout, the perception of family support and depression in nursing personnel.

## Methods

The study was performed in one of the largest hospitals in Greece. Eighteen men and 82 women of the hospital's nursing staff of 670 were selected randomly; 17 men and 78 women agreed to participate. Mean age ± SD was 36.7 ± 6.5 years, with mean work experience 12.9 ± 6.7 years (Table 1). The nurses were asked to give answers to questionnaires for professional burnout, depression, alexithymia and sense of family support; all the subjects did so within 20 minutes at most.

**Table 1:** Descriptive statistics

	**N**	**Minimum**	**Maximum**	**Mean**	**Std. Deviation**
Age	95	21	55	36,612	6,760

Work experience (years)	95	1	31	12,893	6,927

BDI (Depression)	95	0	33	8,473	6,573

FS (Family support)	95	28	65	47,708	9,468

TAS (Alexithymia)	95	20	81	46,842	13,375

E.E (Emotional exhaustion)	95	6	52	26,336	11,655

SPA (Sense of personal accomplishment)	95	7	48	33,778	8,511

DEP (Depersonalization)	95	0	26	10,831	6,365

Alexithymia was assessed with the 20-item self-answered Toronto Alexithymia Scale (TAS-20) [19-21]. Each item is rated from 1 to 5. The questionnaire's translation into modern Greek had good reliability (alpha = 0.80). Scores > 60 indicate alexithymic characteristics [1]. The TAS-20 consists of three factor scales: Difficulty Identifying Feelings (DIF), Difficulty Describing Feelings (DDF) and Externally Oriented Thinking (EOT). The scale includes five negatively keyed items (items 4, 5, 10, 18 and 19).

To measure professional burnout we used Maslach's 22-item Burnout Inventory (MBI), which assesses emotional exhaustion (for evaluation of the frequency of emotional stress due to work), depersonalization (that reflects reactions of indifference and impersonal handling of patients) and the lack of personal achievement (that measures the sense of sufficiency, efficiency and achievements in the professional sector). Each item was answered on a scale from 0 (never) to 6 (every day). The questionnaire was translated into modern Greek and validated with Greek nurses; emotional exhaustion is associated with values higher than 30, depersonalization is associated with levels higher than 11 and lack of personal achievement with values higher than 35, in the relevant subscales [22].

For depression, Beck's Depression Inventory (BDI) was used. It assesses depression through answers in 21 items (rated on a scale from 0 to 3) [23,24]. Its internal validity is high and its test-retest reliability is 0.48-0.86 for clinical and 0.60-0.90 for non-clinical subjects, respectively. External validity vis-à-vis a clinical diagnosis of depression is considered to be satisfactory; scores ≥ 14 indicate moderate to severe depression.

To measure perception of family support we used, as in previous studies, Julkunen's 13-item questionnaire [6,15,25,26]. All 13 items of the scale (e.g. item 1: "My family supports me in all my efforts" and item 5: "I am always the one to blame when our home is a mess") are rated on a five-point scale; scores > 37 indicate an increased sense of family support. The questionnaire's adaptation in modern Greek presented good reliability (alpha = 0.80). Individuals living by themselves (16 of the total sample) did not answer the questionnaire.

Student's t-test, Pearson's correlation and stepwise linear regression were used for the evaluation of data. Two-tailed statistical significance was set at p ≤ 0.05. The computations were carried out with SPSS for Windows, version 15.0, statistical software.

The study was briefly explained to the participants. The confidentiality of the participants' answers was guaranteed. No financial support was necessary.

## Results

All distributions of the continuous variables were normal (One-Sample Kolmogorov-Smirnov Test, p > 0.05).

No statistically significant difference was noted between men and women regarding age (men 35.06 ± 3.27 versus women 37.03 ± 7.39 years, t-test p > 0.05); women, however, had more work experience compared with men (men 8.06 ± 4.16 versus women 13.96 ± 6.98 years, t-test p < 0.01). The questionnaires' scores did not correlate with age; work experience was correlated with BDI scores (Pearson correlation p < 0.05). This correlation disappeared after controlling for age (partial correlation p > 0.05).

Moderate to severe depressive symptoms, with BDI scores > 14 were found in 16.8% of subjects. Alexithymic characteristics, with TAS scores > 60 were noted in 14.7% of subjects, whereas low sense of family support was seen in 15.2% of them. Regarding professional burnout, emotional exhaustion, depersonalization and lack of personal achievement were noted in 38.9%, 46.3% and 49.5% of subjects, respectively; only 14.7% of them had pathological scores in all subscales. Women had higher BDI and TAS-20 scores compared with men (t-test p < 0.05). Women had lower scores than men in the personal achievement subscale of MBI (t-test p < 0.05), as well as in their perception of family support (Table 2).

**Table 2:** Means and differences in BDI, FS, TAS and MBI dimensions between genders

		**N**	**Mean**	**Std. Deviation**	**Std. Error**
BDI (Depression)	MALE	17	6,53*	4,32	0,105
	
	FEMALE	78	8,90*	6,92	0,780

FS (family support)	MALE	15	52,80**	8,04	2,07
	
	FEMALE	64	46,52**	9,43	1,18

TAS (alexithymia)	MALE	17	41,00*	12,06	2,92
	
	FEMALE	78	48,12*	13,38	1,51

E.E (Emotional exhaustion)	MALE	17	28,71	13,21	3,20
	
	FEMALE	78	25,82	11,31	1,28

SPA (Sense of personal accomplishment)	MALE	17	36,58*	6,56	1,59
	
	FEMALE	78	33,17*	8,80	0,997

DEP (Depersonalization)	MALE	17	11,12	7,17	1,74
	
	FEMALE	78	10,77	6,22	0,70

*T test p < 0.05, **T test p < 0.01

Alexithymia was correlated positively with depression, emotional exhaustion and depersonalization and negatively with sense of family support and personal achievement (Table 3). Family support was correlated positively with personal achievement and negatively with depression (Table 3). Controlling for alexithymia, these correlations did not reach statistical significance (Table 4).

**Table 3:** Pearson correlations

		**TAS**	**SE**	**SPA**	**DEP**	**BDI**
	
Pearson correlation Sig. (2-tailed) N = 95	SE	0,361**				
	
	EPE	-0,415**	-0,332**			
	
	AP	0,346**	0,493**	-0,404**		
	
	BDI	0,514**	0,482**	-0,303**	0,367**	
	
	FS	-0,497**	-0,178	0,381**	-0,108	-0,352**

*p < 0.05, **p < 0.01

**Table 4:** Partial correlations

		**SE**	**SPA**	**DEP**	**BDI**
	
Partial Correlation Controlling For Alexithymia (TAS)	EPE	-0,119			
	
	AP	0,442*	-0,238**		
	
	BDI	0,414**	-0,168	0,250*	
	
	FS	-0,001	0,184	0,104	-0,116

*p < 0.05, **p < 0.01

Stepwise linear regression showed that 28.9% and 12.2% of depression values were attributed to alexithymia and emotional exhaustion, respectively. Alexithymia accounted for 24.7% of family support values. Emotional exhaustion was accounted-for mostly by depersonalization (27%) and depression (11.5%). Emotional exhaustion was attributed to 27% of depersonalization; the latter was attributed at 6.2% to personal achievement. Personal achievement was attributed to alexithymia (23.6%) and to depersonalization (4.3%).

## Discussion

There is an ongoing scientific debate regarding the stable or dependent characteristics of alexithymic symptoms, particularly vis-à-vis the relationship between alexithymia and depression [8,18]. Our results lend credence to the relationship between alexithymia and depression, without looking into the above-mentioned debate, which is beyond this study's scope.

Additionally, our findings are in accordance to a previous study [18], which concluded that both alexithymia and depression are associated with burnout, even though they suggest that alexithymia may be an independent risk factor for burnout. Furthermore, it seems that subjects with depressive characteristics show increased vulnerability to burnout because of their innate lack of ability to derive satisfaction from their work [13].

That family support correlates positively with personal achievement is in accordance with previous studies [13]. In these studies, it appeared that increased engagement in family support acts protectively against the development of burnout. On the other hand, it seems possible that individuals with alexithymic characteristics might be unable to benefit from their family support.

This study has limitations; in particular, the workplace conditions per se were not quantified or included in the study's parameters. The inclusion of such parameters is warranted in further studies.

## Conclusion

We tried to interpret the associations of alexithymia with professional burnout, depressive symptoms and family support. Alexithymia was directly associated with depression and personal achievement, but also - indirectly - with the sense of family support.

Although we did not evaluate the effect of specific interventions (such as support groups or family therapy techniques) in individuals with overtly alexithymic characteristics, we believe that alexithymia should be taken into account in interventions targeting depression and/or burnout.

## Competing interests

The authors declare that they have no competing interests.

## Authors' contributions

DB and AT conceived the paper, carried out the mathematical analysis and drafted the paper; CS, AM and KG performed the clinical measurements, collected data and helped draft the manuscript; EZ carried out the mathematical analysis; II conceived the paper, carried out the mathematical analysis and helped draft the paper. All authors read and approved the final manuscript.
